# Left Ventricular Unloading in Patients on Venoarterial Extracorporeal Membrane Oxygenation Therapy in Cardiogenic Shock: Prophylactic Versus Bail-Out Strategy

**DOI:** 10.3390/life13020582

**Published:** 2023-02-19

**Authors:** Darko Radakovic, Armin Zittermann, Sebastian V. Rojas, Dragan Opacic, Artyom Razumov, Emir Prashovikj, Henrik Fox, René Schramm, Michiel Morshuis, Volker Rudolph, Jan Gummert, Christian Flottmann, Marcus-André Deutsch

**Affiliations:** 1 Clinic for Thoracic and Cardiovascular Surgery, Heart and Diabetes Center NRW, Ruhr-University Bochum, 32545 Bad Oeynhausen, Germany; 2 Clinic for General and Interventional Cardiology/Angiology, Heart and Diabetes Center NRW, Ruhr-University Bochum, 32545 Bad Oeynhausen, Germany; 3 Lukas Krankenhaus Bünde Medizinische Klinik II—Innere Medizin und Kardiologie Bünde Germany, 32257 Bünde, Germany

**Keywords:** cardiogenic shock, venoarterial extracorporeal membrane oxygenation (VA ECMO), left ventricular unloading, Impella device

## Abstract

Background: The benefit of prophylactic left ventricular (LV) unloading during venoarterial extracorporeal membrane oxygenation (VA-ECMO) in selected patients at risk of developing LV distension remains unclear. Methods: We enrolled 136 patients treated with Impella pump decompression during VA-ECMO therapy for refractory cardiogenic shock. Patients were stratified by specific indication for LV unloading in the prophylactic vs. bail-out group. The bail-out unloading strategy was utilized to treat LV distension in VA-ECMO afterload-associated complications. The primary endpoint was all-cause 30-day mortality after VA-ECMO implantation. The secondary endpoint was successful myocardial recovery, transition to durable mechanical circulatory support (MCS), or heart transplantation. Results: After propensity score matching, prophylactic unloading was associated with a significantly lower 30-day mortality risk (risk ratio 0.38, 95% confidence interval 0.23–0.62, and *p* < 0.001) and a higher probability of myocardial recovery (risk ratio 2.9, 95% confidence interval 1.48–4.54, and *p* = 0.001) compared with the bail-out strategy. Heart transplantation or durable MCS did not differ significantly between groups. Conclusions: Prophylactic unloading compared with the bail-out strategy may improve clinical outcomes in selected patients on VA-ECMO. Nevertheless, randomized trials are needed to validate these results.

## 1. Introduction

Cardiogenic shock (CS) is a high-acuity, etiologically diverse low cardiac output state resulting in life-threatening end-organ hypoperfusion that is frequently associated with multisystem organ failure [1]. In refractory CS with life-threatening hypotension, temporary mechanical circulatory support (MCS) is recommended, without any preference for device selection to establish end-organ perfusion in order to reverse acidosis and multiple organ failure [2]. Device selection should be guided by clinical judgment, experience, technical expertise, and availability. The European Society of Cardiology (ESC) guidelines recommend using extracorporeal membrane oxygenation (ECMO) or the Impella device for hemodynamic stabilization until other therapeutic options, including heart transplant or long-term MSC, can be evaluated and contraindications for its use excluded (e.g., brain damage after resuscitation) [3]. Venoarterial (VA) ECMO provides full cardiopulmonary support in patients with refractory CS and is currently the most widely used form of temporary MCS [4]. However, retrograde blood flow in the ascending aorta after the cannulation of a peripheral artery increases left ventricular (LV) afterload that may impair heart recovery [5]. Therefore, decompression of the left ventricle is crucial in the setting of severe myocardial dysfunction with increased LV end-diastolic pressure and pulmonary congestion [3,6]. Although various strategies to decompress a left ventricle have been described [7,8], active unloading with an Impella percutaneous ventricular assist device has been reported to improve survival [9,10,11]. Nonetheless, adding a second device in VA-ECMO patients can increase the already high risk of complications, such as bleeding, infection, hemolysis, and renal failure [12]. Thus, patient-specific factors should be considered before initiating active unloading therapy upfront as the prophylactic strategy in all patients on VA ECMO support. In contrast, in the bail-out approach LV unloading is implemented post-hoc to treat the consequences of clinically or hemodynamically apparent LV distension [13]. Utilization of these two strategies may have different effects on patient prognosis, warranting further analyses [14]. 

Therefore, the present study sought to determine the clinical outcomes of patients undergoing concomitant VA-ECMO and active LV unloading Impella therapy with a specific focus on the impact of the two different strategies in these patients. 

## 2. Materials and Methods

### 2.1. Study Design and Patients

**Figure 1:**
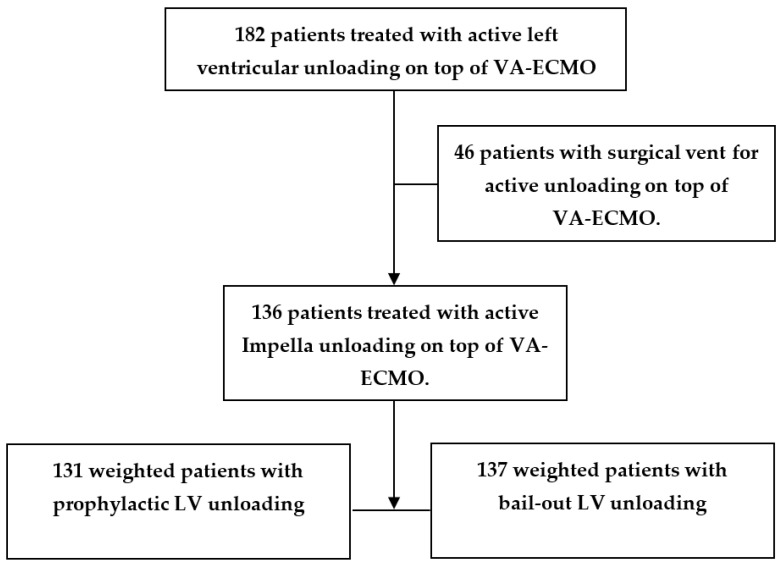
Study flow diagram. VA-ECMO: venoarterial extracorporeal membrane oxygenation therapy.

We conducted a retrospective analysis of the prospective clinical database. All patients with VA-ECMO and concomitant active LV unloading between January 2014 and June 2021 were included (Figure 1). Patients with surgical LV vent as the unloading modality were excluded from further analysis. The remaining 136 VA-ECMO patients with concomitant active Impella CP (ABIOMED Inc, Danvers, MA, USA) LV unloading were reviewed. The specific technical aspects of device implantation were observed in accordance with the relevant guidelines, as have been previously described [7]. Briefly, a centrifugal pump console together with an oxygenator and a heat exchanger in the standard ECMO configuration were used. Arterial cannulation was performed after heparin administration via the femoral artery either surgically or through the Seldinger technique. Venous drainage was initiated under the guidance of transesophageal echocardiography for adequate placement of the distal venous cannula in the right atrium. After the activated clotting time goal (160–180 s) was achieved, ECMO support was started with subsequent daily monitoring. Bedside surveillance and regular circuit checks were part of regular patient management. These checks also included documentation of pump flow parameters, ECMO gas blender setting, as well as patient right radial artery blood gas analysis. Maintaining pulsatile arterial pressure with mean values above 60 mm Hg was targeted. Assessment of LV decompression was performed by means of hemodynamic monitoring and echocardiography evaluation. If possible, satisfactory LV loading conditions and contractility as well as adequate cardiac rhythm were sought.

Thereafter, an interdisciplinary team decided to initiate prophylactic unloading therapy in selected patients thought to be at a higher risk of developing LV distension. This included patients who were expected to stay in persistent hemodynamic or respiratory instability related to impaired LV unloading, cases in which afterload reduction could not be achieved, or if inotropic support was not justified (e.g., in the setting of myocardial ischemia or infarction). Beyond this, the team also considered other clinical factors, such as the severity of ventricular dysfunction, underlying heart pathology, and the absence of reversible causes of myocardial dysfunction. Furthermore, serial measurements of left-sided filling pressures as surrogates of the LV preload and its diastolic operating compliance [15] were evaluated. 

A post-hoc bail-out approach was used to treat LV distension in patients on VA-ECMO with clinical criteria of inadequate LV unloading, such as pulmonary edema, blood stasis seen in echocardiography, or absent ejection despite advanced management and monitoring. 

In our institution, Impella is a first-choice unloading modality for patients on VA-ECMO, assuming potential contraindications for its usage (mechanical aortic valve, peripheral artery disease, or LV thrombus) are excluded. After careful assessment of heart loading conditions and contractility reserve, Impella CP was implanted via femoral artery access under fluoroscopic guidance in order to confirm correct and stable placement. Bi-lateral distal perfusion 5F catheters were inserted into superficial femoral arteries in patients with malperfusion of the lower extremities. Clinical assessment included rigorous echocardiography-based trials with flow reductions in active unloading therapy in order to identify compensated patients. Hemodynamic stability on minimal pharmacologic support as assessed by vasoactive inotropic score, pulsatile arterial pressure waveform with a pulse pressure above 20 mmHg coupled with stable laboratory surrogates of end-organ function or failure, and maintained cardiac output at P2 Impella pump speed were fundamental before considering the weaning trial. Patients with serial weaning failures were evaluated for durable ventricular assist device implantation [7].

### 2.2. Data Acquisition

The model for end-stage liver disease score, sepsis-related organ failure assessment (SOFA) score and acute physiology and chronic health evaluation (APACHE) 2 score were calculated after intensive care admission. Survival after venoarterial ECMO (SAVE) score calculations were based on pre-implantation data. Maximum Vasoactive Inotropic Score (defined as dopamine dose (µg/kg/min) × 1 + dobutamine dose (µg/kg/min) × 1 + adrenaline dose (µg/kg/min) × 100 + noradrenaline dose (µg/kg/min) × 100 + phenylephrine dose (µg/kg/min) × 100) and blood lactate levels were recorded as the maximum values in the 6 h prior to VA-ECMO cannulation. Baseline and demographic data (age, sex, body mass index, diabetes mellitus, resuscitation, and chronic kidney disease) as well as outcome variables were retrospectively acquired from case histories (Table 1). The study was approved by the ethics committee of Ruhr university Bochum (approval: 2021-761; 19 February 2021).

### 2.3. Endpoints

The primary endpoint was all-cause 30-day mortality after VA-ECMO implantation. Secondary endpoints were successful myocardial recovery, transition to durable MCS, or heart transplantation. Additionally, missing data were collected via interviews and anonymized. Safety endpoints included cannulation site bleeding, hemolysis, sepsis defined as objective signs of infection with persisting systemic inflammatory response syndrome and >2 positive blood samples, stroke (confirmed by computed tomography), intervention due to access-site-related ischemia, and abdominal complications. 

### 2.4. Statistical Analyses 

Propensity scores (PSs) were calculated because of the nonrandomized group allocation. To create the PS for each patient, the team used the multivariable logistic regression model with the type of treatment (prophylactic or bail-out) as the binary dependent variable. The model comprised the following baseline covariates: age, sex, body mass index, diabetes mellitus, pulmonary hypertension, resuscitation, estimated glomerular filtration rate (eGFR), chronic kidney disease, total bilirubin, and Maximum Vasoactive Inotropic Score. All these variables were included regardless of statistical significance. After the PS was established, inverse probability of treatment weighting (IPTW) was applied to reduce the bias of unweighted estimators and adjust for covariate imbalance between the two study groups. The following formula was applied: T/PS + (1 − T)/(1 − PS), where T indicates patient status (0 in patients with the bail-out treatment or 1 in patients with the prophylactic treatment). Standardized mean differences (SMDs) were used to evaluate the post-weighting balance in covariates. The balance was considered satisfactory when the SMD was less than 10%. To calculate SMD, all continuous parameters in Table 1 are presented as mean with standard deviation (SD). Since age, PS, and time interval between Impella and VA-ECMO implantation were nonnormally distributed, as assessed by the Kolmogorov–Smirnov test, we present these data in the text as median with interquartile ranges. The Mann–Whitney U test was applied to test for differences in continuous data between groups accordingly. For time-to-event analyses, Kaplan–Meier non-parametric estimates of event-free survival were created for two study groups. Fisher’s exact test was used to compare IPTW-adjusted clinical outcome parameters, such as 30-day mortality (primary endpoint), successful myocardial recovery, heart transplantations or VAD (secondary endpoints), cannulation site bleeding, stroke, hemolysis, interventions due to access-site-related ischemia, abdominal complication, and sepsis (safety endpoints).

Clinical outcome data are presented as relative risks (RRs) and 95% confidence intervals (CIs), with *p*-values < 0.05 being considered statistically significant. The software package IBM SPSS, Version 24 (IBM Corp, Armonk, NY, USA) was used for the statistical analysis. Additionally, the PSMATCHING3 R Extension 4 command (Version 2.15.3, R Core Foundation, Austria, Vienna) was added as an SPSS extension bundle under the SPE file format to be able to run this extra program feature in SPSS.

## 3. Results

### 3.1. Patient Characteristics

**Figure 2:**
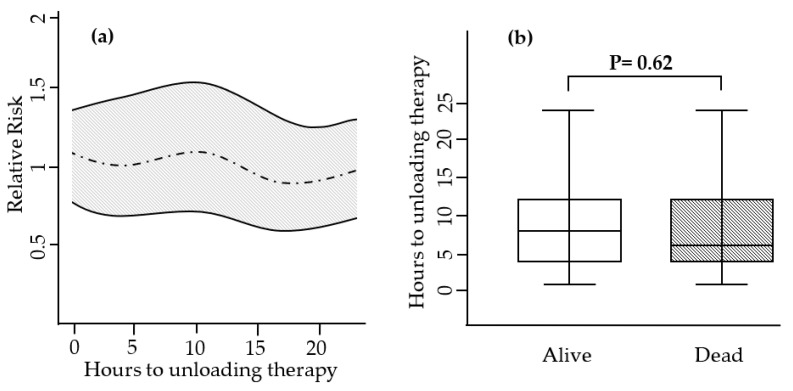
(**a**) The y-axis displays the time interval in hours to active LV unloading therapy after VA-ECMO implantation in hours. The x-axis displays the relative risk for 30-day mortality. (**b**) Boxplots comparing time in hours to active LV unloading therapy among alive and dead patients.

Our cohort comprised a total of 136 patients with refractory CS treated with an Impella LV unloading during VA-ECMO therapy. The median age of the cohort was 58 years (IQR 65-49 years), and 78 patients (57%) with CS suffered a cardiac arrest. The bail-out group consisted of 90 patients (patients with LV distention) who received post-hoc bail-out Impella unloading. Forty-six patients received prophylactic Impella LV unloading after VA-ECMO initiation without developing clinical signs of LV overload during dual MCS therapy (prophylactic group). The median interval between Impella and VA-ECMO implantation was 6 (IQR 12-4) h. Similar RRs for 30-day mortality were observed for every given time interval (in hours) between VA-ECMO and LV unloading initiation (Figure 2a). Furthermore, there was no difference in the median interval between VA-ECMO and Impella LV unloading between the prophylactic and bail-out groups (median 6 h; IQR 13-4 h vs. median 8 h; IQR 12-4 h; *p* = 0.68), and between alive and dead patients (median 8 h; IQR 12-4 h vs. median 6 h; IQR 12-4 h; *p* = 0.62; Figure 2b).

The baseline characteristics of the unweighted and weighted study groups are shown in Table 1. The PS ranged from 0.13016 to 0.66598. The PS in the unweighted bail-out and prophylactic groups was 0.32337 (IQR: 0.40328-0.25569) and 0.37496 (IQR: 0.45988-0.28873), and in the weighted groups, it was 0.32299 (IQR: 0.40904-0.27480) and 0.32933 (IQR: 0.42794-0.26267), respectively. IPTW substantially reduced the SMD in preoperative covariates between the study groups (Table 1). In the IPTW groups, all standardized differences were <10% with the exception of a slightly higher value regarding the age group <50 years. 

### 3.2. Endpoints 

**Figure 3:**
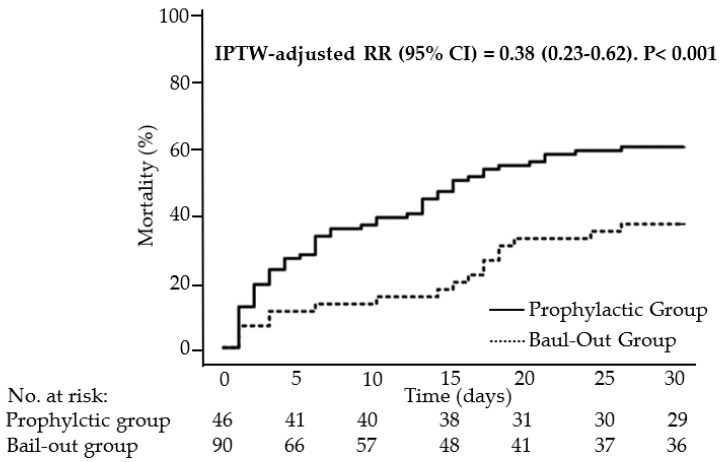
Unadjusted Kaplan–Meier estimates of 30-day mortality by study group and IPTW-adjusted analysis of relative risk with 95% CI for the prophylactic versus bail-out group. CI: confidence interval; IPTW: inverse probability of treatment weighting.

Overall, the 30-day mortality of the cohort was 52%. The most frequent primary modes of death were multiorgan system failure typically associated with sepsis and mesenterial or massive global cerebral ischemia in resuscitated patients. The primary endpoint (30-day mortality) was significantly lower in the prophylactic group than in the bail-out group (36% and 60%, respectively), with an RR of 0.38 (95% CI: 0.23–0.62, and *p* < 0.001, Figure 3). Furthermore, successful myocardial recovery was significantly higher in the prophylactic than in the bail-out group (37% and 18%, respectively), with an RR of 2.9 (95% CI: 1.48–4.54, and *p* = 0.001), whereas heart transplantation or durable MCS did not differ significantly between groups. Patients with myocardial recovery had a VA-ECMO flow rate usually between 1.2 and 1.5 L/min after unloading therapy was discontinued. In patients who recovered, Impella was weaned after median support of 6 (IQR 9-4) days, and VA-ECMO after median of 14 (21-12) days. Prolonged VA-ECMO therapy was needed in patients with protracted desaturation due to congestion caused by severe CS.

Regarding safety endpoints, the risk of stroke was significantly lower in the prophylactic than in the bail-out group (RR = 0.39 (95% CI: 0.19–0.74), *p* = 0.006), as was the risk of hemolysis (RR = 0.51 (95% CI: 0.30–0.86) *p* = 0.012). Other safety endpoints, such as cannulation site bleeding, interventions due to access-site-related ischemia, abdominal complications, and sepsis, did not differ significantly between study groups. Endpoints are presented for the weighted study groups in Table 2.

## 4. Discussion

In this study, prophylactic LV unloading was associated with a significantly lower relative risk of 30-day mortality than bail-out unloading. Furthermore, the prophylactic group exhibited higher rates of myocardial recovery and a significantly lower risk of he-molysis and stroke than the bail-out group.

CS continues to be associated with significant mortality and morbidity irrespective of its underlying etiology [1]. At present, VA-ECMO is the most commonly used device to provide temporary MCS in patients with refractory CS [2]. An important limitation of this strategy is a resultant increase in LV afterload, which can cause pulmonary congestion, LV distension, delayed ventricular recovery, or intracardiac thrombus formation [16]. Therefore, assessment of arterial waveform pulsatility, pulmonary artery occlusion, and diastolic pressures should represent the standard of care. Next, examination of blood gas data may also be particularly useful for diagnosing differential hypoxia, which is consistent with LV distension. Pharmacological treatment to achieve adequate LV loading conditions and contractility as well as satisfactory cardiac rhythm coupled with pump flow reduction may be beneficial for LV compliance during VA-ECMO support in some patients. Furthermore, hemofiltration or diuretics may be used for volume optimization before the utilization of an invasive LV unloading approach. Several different methods have been described for invasive unloading approaches, and device choice is mostly guided by clinical experience, technical expertise, and availability [14]. For example, recent ESC 2021 guidelines recommend LV decompression with a ventricular vent or Impella pump in VA-ECMO patients with increased LV end-diastolic pressure and pulmonary congestion [3]. However, the impact of an active unloading strategy must be further evaluated because various devices can generate different results [7,17]. Positive effects on ventricular decompression and coronary circulation may be achieved with an intra-aortic balloon pump (IABP). Although its routine use remains questionable according to the current guidelines [3], when used as an unloading modality on top of VA-ECMO, it increased LV compliance significantly compared with isolated VA-ECMO-supported patients [8]. Moreover, adding IABP as an unloading strategy offered better 30-day survival and higher weaning rates compared with VA-ECMO therapy only. However, the question remains if these results were influenced by selection bias due the fact that IABP therapy was initiated before VA-ECMO in most cases. In a recent study [7], we found that an Impella unloading strategy was associated not only with more pronounced hemodynamic stabilization, but also higher rates of myocardial recovery compared with surgical LV vent unloading. These results indicate that effective LV decompression may only be achieved with devices that are able to provide several liters of blood flow and thereby better coronary perfusion. Despite the fact that several studies suggest that active LV Impella unloading in VA-ECMO patients may improve survival, adding a second device increases complications (ischemic complications, bleeding, abdominal compartment, and renal failure) [9,12]. Consequently, it remains unclear whether LV unloading should be utilized prophylactically in all patients on VA-ECMO support or only in selected patients at a higher risk of developing LV distension.

In a large international multicenter cohort study, Schrage et al. [12] suggested that early active unloading (e.g., Impella implantation before or within 2 h of the VA-ECMO) was associated with lower 30-day mortality compared with VA-ECMO alone. Moreover, another recent study observed an increasing mortality risk with every extra hour of delay of active LV unloading [18]. Although both studies carried potential indication bias (VA-ECMO represented escalation therapy in most patients), LV decompression in the first 2 h of disease course appeared to prevent irreversible myocardial injury before an LV overload became clinically apparent [12,18]. Once these complications (LV distention, heart without obvious ejection, closed aortic valve, ventricular stasis, or uncontrolled pulmonary edema) occur, they may further impair survival. In the present study, there was no significant difference between the two groups in the timing of initiation of active LV Impella unloading after VA-ECMO with a median 6-h interval between device implantations. This finding suggests that strict cutoffs for prophylactic unloading should be further evaluated since afterload-associated complications may occur at any time due to the complex VA-ECMO-associated afterload pathophysiology. The individual left ventricle load characteristics depend on the metabolic factors, the extent of the myocardial infarction, residual LV contractility, the filling status of the ventricle, and ECMO-related afterload [19]. A sudden excessive increase in afterload may result in a dramatic rise in LV end-diastolic pressure. Subsequently, through an increase in the ventriculoatrial gradient, the left atrial and pulmonary capillary wedge pressures (PCWPs) rise, causing pulmonary edema. The LV distends, further increasing wall tension and myocardial oxygen demand [5]. A vicious cycle develops, further deteriorating cardiac output and finally leading to a nonejecting heart [6,20,21,22]. At this point, bail-out unloading may not be sufficient to reverse circulatory failure and improve the outcome. Identifying patients at risk through strict monitoring of the left-sided filling pressures (e.g., via PCWP or echocardiography), adequate right-sided cardiac decompression, contractility, and heart rhythm is of paramount importance because even after longer periods of VA-ECMO support, patients may benefit from prophylactic unloading.

Furthermore, prophylactic unloading was associated with higher rates of myocardial recovery compared with the bail-out strategy. This association appears logical because the severity of LV distension is inversely correlated with the probability of myocardial recovery [23,24]. It seems that myocardial recovery is more difficult with the post-hoc bail-out strategy to treat distension in VA-ECMO patients with increased myocardial wall stress and higher myocardial oxygen demands. However, the bail-out approach may be considered in certain patients as a bridge to durable MCS.

Lastly, patients treated with a prophylactic LV decompression strategy may be less likely to develop stroke-inducing thrombi than patients with VA-ECMO-associated blood stasis in the aortic root and potential thrombus formation [25,26]. Of note, no difference in vascular complication rates between these two groups was found, and the results were comparable to those of previous studies [9,27,28,29,30]. Prospective randomized trials are required to confirm these promising results.

### Limitations

This study has some limitations. For instance, this work was a nonrandomized, observational, single-center retrospective study, and even after adjustment for several confounders, the generalizability of the results is limited. Although careful documentation of the unloading strategy and its indication facilitated this analysis, choosing the best LV unloading strategy was based on specific clinical scenarios and may have caused a selection bias. The associated patient status in this analysis reflected their status at the time of data capture and so potentially does not reflect the dynamic LV unloading entity. Invasive hemodynamic parameters or echocardiography measurements were not recorded in a standardized fashion, so they could not be included in this analysis. However, our study can be considered hypothesis-generating for the design of future investigations and adds further evidence to aid interdisciplinary shock teams in the decision-making process before applying the unloading strategy in VA-ECMO patients.

## 5. Conclusions

This study’s data indicated a lower 30-day mortality risk with prophylactic LV unloading than produced by the bail-out approach. Moreover, the prophylactic prevention of VA-ECMO-associated afterload complications was shown to positively modulate myocardial recovery and reduce the probability of stroke.

## Figures and Tables

**Table 1:** Baseline characteristics in unweighted and weighted study population.

Parameter	Prophylactic Group n = 46	Unweighted Patients Bail-Out Group n = 90	SMD %	Prophylactic Group n = 131	Weighted Patients Bail-Out Group n = 137	SMD %
Age Group ^1^						
<50 Years	8 (17)	25 (28)	34.0	28 (21.4)	34 (24.8)	10.9
50–60 Years	16 (35)	34 (38)	8.6	51 (38.9)	51 (37.2)	−4.9
>60 Years	22 (48)	31 (34)	−41.2	52 (38.7)	52 (38.0)	−2.0
Sex, Males ^2^	34 (74)	65 (72)	−6.3	96 (73.3)	99 (72.8)	−1.6
Body Mass Index (kg/m^2^) ^1^						
<20	4 (9)	7 (8)	−4.9	10 (7.6)	11 (8.0)	2.0
20–30	30 (65)	62 (69)	12.1	89 (67.9)	94 (68.6)	2.1
>30	12 (26)	21 (23)	−9.9	32 (24.4)	32 (23.4)	−3.3
Diabetes Mellitus ^1^	12 (26)	20 (22)	−13.4	35 (26.7)	34 (24.8)	−6.1
Pulmonary Hypertension ^1^	1 (2)	5 (6)	22.1	4 (3.1)	6 (4.4)	8.1
Resuscitation ^1^	23 (50)	54 (60)	28.6	70 (56.9)	78 (53.4)	−9.8
Chronic Kidney Disease ^1^	7 (15)	12 (13)	−8.1	17 (13.0)	18 (13.1)	0.4
eGFR Group (mL/min/1.73m^2^) ^1^						
<30	18 (39)	27 (30)	−27.3	45 (34.4)	45 (32.8)	−4.7
30–60	21 (46)	40 (44)	−5.6	60 (45.8)	60 (44.5)	−3.7
>60	7 (15)	23 (26)	34.8	26 (19.8)	31 (22.6)	9.3
Total Bilirubin > 1.2 mg/dL ^1^	22 (48)	50 (56)	22.6	70 (53.4)	72 (52.6)	−2.2
Maximum Vasoactive Inotropic Score ^2^	40.9 (22.4)	42.5 (22.7)	7.1	41.9 (21.9)	42.5 (22.4)	2.7
SOFA Score ^2^	10.8 (3.7)	10.6 (5.4)	−4.3	10.8 (3.7)	10.5 (4.8)	−7.0
APACHE II Score ^2^	26.6 (7.8)	26.3 (10.4)	−3.3	26.7 (8.0)	26.8 (10.0)	−1.1
SAVE Score ^2^	−10.8 (4.6)	−10.4 (4.5)	−8.8	−10.7 (4.6)	−10.3 (4.6)	−8.7
Serum Lactate (mmol/L) ^2^	10.9 (5.2)	10.1 (5.2)	−15.4	10.9 (5.3)	10.4 (5.4)	−9.3

^1^ number and percentage; ^2^ mean and standard deviation. Abbreviations: eGFR: estimated glomerular filtration rate; SMD: standardized mean difference.

**Table 2:** Clinical outcomes in the IPTW groups.

	Prophylactic Group n = 131	Bail-Out Group n = 137	Relative Risk (95%CI)	*p*-Value
**Primary Endpoint**				
Thirty-Day Mortality (n,%)	47 (35.9)	82 (59.9)	0.38 (0.23–0.62)	<0.001
**Secondary Endpoints**				
Myocardial Recovery (n,%)	48 (36.6)	25 (18.2)	2.9 (1.48–4.54)	0.001
Heart Transplantation or VAD (n,%)	30 (22.9)	33 (24.1)	0.94 (0.53–1.65)	0.89
**Safety Endpoints**				
Cannulation Site Bleeding (n,%)	10 (7.6)	19 (13.9)	0.51 (0.23–1.15)	0.12
Stroke (n,%)	14 (10.7)	33 (24.1)	0.39 (0.19–0.74)	0.006
Hemolysis (n,%)	31 (23.7)	52 (38.0)	0.51 (0.30–0.86)	0.012
Access-Site-Related Ischemia (n,%)	14 (10.8)	20 (14.7)	0.70 (0.34–1.45)	0.36
Abdominal Complications (n,%)	45 (34.4)	48 (35.0)	0.97 (0.59–1.61)	>0.99
Sepsis (n,%)	49 (37.7)	59 (43.1)	0.80 (0.49–1.31)	0.39

Abbreviations: IPTW: inverse probability of treatment weighting; n: number; VAD: ventricular assist device; CI: confidence interval.

## Data Availability

The data underlying this article will be shared on reasonable request to the corresponding author.
